# Functional Characterization of a Flavone Synthase That Participates in a Kumquat Flavone Metabolon

**DOI:** 10.3389/fpls.2022.826780

**Published:** 2022-03-02

**Authors:** Shulin Tian, Yuyan Yang, Tao Wu, Chuan Luo, Xin Li, Xijuan Zhao, Wanpeng Xi, Xiaogang Liu, Ming Zeng

**Affiliations:** ^1^College of Horticulture and Landscape Architecture, Southwest University, Chongqing, China; ^2^Key Laboratory of Horticulture Science for Southern Mountainous Regions, Ministry of Education, Chongqing, China

**Keywords:** kumquat plants, flavonoids, flavone synthase, protein–protein interaction, metabolon

## Abstract

Flavones predominantly accumulate as *O*- and *C*-glycosides in kumquat plants. Two catalytic mechanisms of flavone synthase II (FNSII) support the biosynthesis of glycosyl flavones, one involving flavanone 2-hydroxylase (which generates 2-hydroxyflavanones for *C*-glycosylation) and another involving the direct catalysis of flavanones to flavones for *O*-glycosylation. However, FNSII has not yet been characterized in kumquats. In this study, we identified two kumquat *FNSII* genes (*FcFNSII-1* and *FcFNSII-2*), based on transcriptome and bioinformatics analysis. Data from *in vivo* and *in vitro* assays showed that FcFNSII-2 directly synthesized apigenin and acacetin from naringenin and isosakuranetin, respectively, whereas *FcFNSII-1* showed no detectable catalytic activities with flavanones. In agreement, transient overexpression of *FcFNSII-2* in kumquat peels significantly enhanced the transcription of structural genes of the flavonoid-biosynthesis pathway and the accumulation of several *O*-glycosyl flavones. Moreover, studying the subcellular localizations of FcFNSII-1 and FcFNSII-2 demonstrated that N-terminal membrane-spanning domains were necessary to ensure endoplasmic reticulum localization and anchoring. Protein–protein interaction analyses, using the split-ubiquitin yeast two-hybrid system and bimolecular fluorescence-complementation assays, revealed that FcFNSII-2 interacted with chalcone synthase 1, chalcone synthase 2, and chalcone isomerase-like proteins. The results provide strong evidence that FcFNSII-2 serves as a nucleation site for an *O*-glycosyl flavone metabolon that channels flavanones for *O*-glycosyl flavone biosynthesis in kumquat fruits. They have implications for guiding genetic engineering efforts aimed at enhancing the composition of bioactive flavonoids in kumquat fruits.

## Introduction

Flavones, one of the largest subclasses of flavonoids, perform various physiological roles in plants, such as participating in responses to biotic and abiotic stresses (Morimoto et al., 1998; Du et al., 2010a,b; Kong et al., 2010; Jiang et al., 2016; Righini et al., 2019). In addition, these metabolites contribute to the internal and external qualities of fruits, herbs, and vegetables by improving their appearance, taste, and nutritional value. Kumquats look like miniature oranges, are consumed worldwide, and contain diverse and abundant bioactive flavonoids. Kumquat extract possesses several health-promoting effects, and these effects are associated with the compositions and quantities of these flavonoids (Sadek et al., 2009; Barreca et al., 2014; Lou et al., 2015; Nagahama et al., 2015). In contrast to the many other fruits that tend to biosynthesize anthocyanins and flavonols, kumquats and other citrus fruits predominantly accumulate large amounts of flavanones and flavone derivatives (Montanari et al., 1998; Lou et al., 2015; Butelli et al., 2017; Wang Y. et al., 2017; Zhao et al., 2020).

Flavonoid biosynthesis in higher plants initiates from the stepwise condensation of *p*-coumaroyl-coenzyme A (CoA) with three malonyl-CoAs in a reaction catalyzed by chalcone synthase (CHS). This is followed by chalcone cyclization into naringenin by chalcone isomerase (CHI) (Winkel-Shirley, 2001). Flavanone naringenin is a biochemical precursor used for the biosynthesis of many subclasses of flavonoids such as flavonols, anthocyanins, and flavones. The biosynthesis of flavones from flavanones in higher plants is catalyzed by two distinct flavone synthases (FNSs), FNSI and FNSII; FNSII is phylogenetically more widespread than FNSI (Jiang et al., 2016). FNSI, is a member of the Fe^2+^/2-oxoglutarate-dependent dioxygenase (2-OGDD) family; it directly generates flavones from flavanones. Early research suggested that FNSI enzymes only occurred in the Apiaceae family (Gebhardt et al., 2005, 2007). Later, FNSI enzymes were identified in rice (Lee et al., 2008), *Arabidopsis*, and maize (Falcone Ferreyra et al., 2015), indicating that FNSI is more widely distributed (Jiang et al., 2016). All the functionally characterized FNSII enzymes are derived from the CYP93G subfamily in monocots and from the CYP93B subfamily in dicots. Similar to FNSI enzymes, most FNSII enzymes catalyze the direct conversion of flavanones into flavones through C2,C3-*cis-*desaturation (Martens and Mithofer, 2005). Interestingly, licorice CYP93B1 (Akashi et al., 1998), maize CYP93G5 (Morohashi et al., 2012), *Medicago truncatula* CYP93B10/11 (Zhang et al., 2007), rice CYP92G2 (Du et al., 2010a), and sorghum CYP93G3 (Du et al., 2010b) function as flavanone 2-hydroxylases (F2Hs) by catalyzing the conversion of flavanones into 2-hydroxyflavanones for *C*-glycosylation.

**FIGURE 1:**
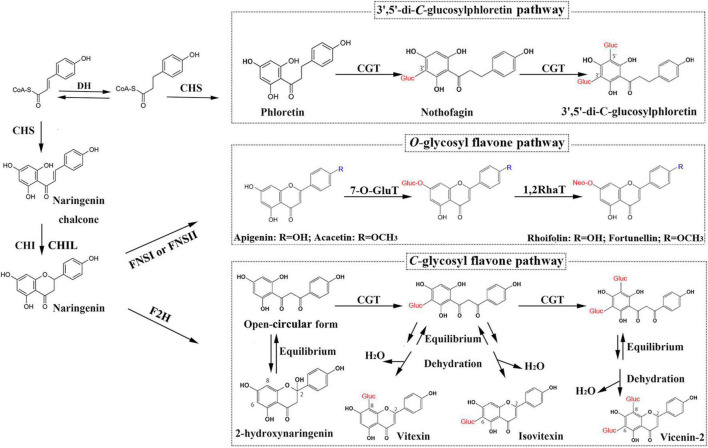
Glycosyl flavone-biosynthesis pathways in kumquat plants. DH, dehydrogenase; CHS, chalcone synthase; CHI, chalcone isomerase; CHIL, chalcone isomerase-like; FNSI, flavone synthase I; FNSII, flavone synthase II; F2H, flavanone 2-hydroxylase; 7-*O*-GluT, flavonoid 7-*O*-glucosyltransferase; 1,2RhaT, 1,2-rhamnosyltransferase; CGT, *C*-glycosyltransferase.

Except for trace amounts of polymethoxylated flavones (PMFs), kumquat flavones predominantly exist as *C*- or *O*-glycosides (Lou et al., 2016; Lou and Ho, 2017; Figure 1): *O*-glycosides such as rhoifolin (RHO) and fortunellin (FOR) and *C*-glucosides such as isovitexin, vitexin, vicenin-2 (VIC), 8-*C*-neohesperidosyl apigenin (APN), and margaritene (MAR). The sugar moieties of the *C*-glycosides are directly linked to the basic flavonoid skeleton through their respective carbon atoms. In *O*-glycosides, the sugar moieties are bonded to the hydroxyl groups of flavonoid aglycones. *C*-glycosylation can occur before the formation of the flavone backbone, whereas *O*-glycosylation usually occurs after backbone formation (Martens and Mithofer, 2005; Wu et al., 2016; Wang X. et al., 2017). A kumquat *C-*glycosyltransferase (CGT) utilizes 2-hydroxyflavanones, rather than flavones, as sugar acceptors and produces the corresponding *C*-glucosides (Ito et al., 2017). Therefore, FNSII enzymes might operate through multiple catalytic mechanisms.

The enzymes necessary for flavonoid biosynthesis could form flavonoid metabolons (ordered protein complexes), which are believed to occur in diverse plant species (Hrazdina and Wagner, 1985; Winkel, 2004; Nakayama et al., 2019). Specific protein–protein interactions could regulate the biosynthetic efficiency and spatiotemporal distributions of flavonoids. Metabolons are anchored to the endoplasmic reticulum (ER) membrane; therefore, ER-resident P450 enzymes could serve as anchors for metabolons. The formation of flavonoid metabolons on ER-resident P450 occurs in multiple plants, including rice (Shih et al., 2008), snapdragon, torenia (Fujino et al., 2018), and soybean (Mameda et al., 2018). In soybean roots, 2-hydroxyisoflavanone synthase (a P450 enzyme) can interact with chalcone reductase (CHR) and CHS to form a flavonoid metabolon, in which GmCHR5 is located near CHS to reduce the transit time of the CHR substrate from CHS to CHR (Mameda et al., 2018). In snapdragon and torenia plants, enzymes involved in late-stage anthocyanin biosynthesis interact with FNSII, although FNSII activity was not necessary for anthocyanin biosynthesis (Fujino et al., 2018). Therefore, ER-bound P450s (such as FNSII) are posited as components of flavonoid metabolons.

Considering that FNSII (also including F2H) are the only P450 enzymes involved in the biosynthetic pathway for *O*/*C*-glycosyl flavones in kumquat fruits (Figure 1), we hypothesized that they likely play roles in anchoring dynamic flavone metabolons to the ER. According to our model, specific protein–protein interactions modulate the diversity in terms of the accumulation patterns and chemical structures of flavone derivatives. However, no FNSII enzymes have been characterized in kumquat plants; this has limited further study on the regulatory and biosynthetic mechanisms of flavones and their derivatives. Therefore, in this study, we isolated 2 *FNSII* genes (*FcFNSII-1* and *FcFNSII-2*) through RNA-seq analysis. The results of *in vivo*, *in vitro*, and *in planta* experiments indicate that *FcFNSII-2* directly converted flavanones into corresponding flavones, whereas *FcFNSII-1* had no detectable catalytic activity against flavanones. Both *FcFNSII-1* and *FcFNSII-2* localized to the ER; however, only the latter interacted with upstream enzymes in the flavonoid-biosynthesis pathway. These findings imply that FcFNSII-2 might serve as a nucleation site for flavone-metabolon formation, mediating the biosynthesis of *O*-glycosyl flavone and its derivatives in kumquats.

## Materials and Methods

### Plant Materials

“Huapi” kumquat trees (*Fortunella crassifolia*) were grown at the Citrus Research Institute (Chongqing, China). The flowers, young leaves, and young shoots were collected in the spring, and the fruits were collected at different growth stages, including 30, 90, and 150 days after full blooming (DAB). The samples were immediately frozen in liquid nitrogen and stored at −80°C until use.

### Isolation and Bioinformatics Analysis of FNSIIs

Total RNA from the fruit peels, flowers, young leaves, and young shoots of “Huapi” trees were extracted using the MiniBEST Plant RNA Extraction Kit (TaKaRa Bio). cDNA was synthesized for reverse transcription-polymerase chain reaction (RT-PCR) analyses using the PrimeScript™ Double Strand cDNA Synthesis Kit (TaKaRa Bio). Subsequently, the open-reading frames (ORFs) of different *FcFNSII*s were cloned using PCR and gene-specific primers (Supplementary Table 1). A phylogenetic tree of the deduced amino acid (AA) sequences was constructed using the MEGA 6.0 program (Tamura et al., 2013), using the neighbor-joining method.

### Quantitative Reverse Transcription-Polymerase Chain Reaction Analysis

Following cDNA synthesis, quantitative PCR was performed using the TB Green^®^ Fast qPCR Mix (TaKaRa, Beijing, China) on a LightCycler 480 real-time system (Roche, Basel, Switzerland). The PCR mix (10 μL) contained 5 μL of 2 × TB Green *Premix Ex Taq* II (Tli RNaseH Plus), 50 ng of cDNA, and 0.4 μM of each primer (Supplementary Table 1). The reaction conditions were: 95°C for 30 s, followed by 35 cycles at 95°C for 5 s, 56–58°C for 10 s, and 72°C for 25 s. *Actin* was used as the internal reference gene for normalizing the Quantitative PCR data. The relative transcript levels were calculated using the 2^–ΔΔCt^ method. Three biological replicates were analyzed for each gene. The sequences of the primers are included in Supplementary Table 1.

### Yeast Expression of Recombinant Proteins and Enzyme Assays

Yeast expression and *in vivo* yeast assays was performed according to previous studies (Lam et al., 2014; Wu et al., 2016; Righini et al., 2019), with several minor modifications. The ORF of each *FcFNSII*s were ligated directly into the yeast expression vector, pESC-HIS (to replace the FLAG epitope) and expressed under the control of the GAL10 promoter. Unless otherwise specified, all recombinant vectors were constructed following a seamless cloning strategy (ClonExpress Ultra One Step Cloning Kit, Vazyme, China). All recombinant plastids and the empty vector (negative control) were separately introduced into the *Saccharomyces cerevisiae* strain WAT11. Briefly, yeast cultures were grown overnight at 30°C in liquid synthetic dextrose minimal medium lacking histidine; they were collected through centrifugation and diluted to an optical density (at 600 nm: OD_600_) of 1.0 in induction medium (synthetic galactose minimal medium). After inducing protein expression for 6 h, the substrate naringenin or isosakuranetin was added to a final concentration of 0.2 mM. Prior to addition, the flavanone substrates were dissolved in dimethyl sulfoxide (DMSO). After a 24 h incubation, the reactions were terminated by extraction with ethyl acetate, evaporated under nitrogen gas, and dissolved in 200 μL of 80% methanol for ultra-high performance liquid chromatography-quadrupole time-of-flight mass spectrometry (UPLC-Q-TOF-MS) analysis.

Yeast microsomes were extracted from the transformed cells, as described previously (Lam et al., 2014; Wu et al., 2016). The microsomal proteins were quantified using the Bradford Protein Assay (TransGen Biotech, Beijing, China). The enzyme activities were assayed at 30°C for 1 h in 100 mM Tris/HCl buffer (pH 7.0) containing 200 μg of microsome protein, 1 mM NADPH, 1 mM diethyl dithiocarbamate, 1 mM dithiothreitol, and appropriate amounts of substrate dissolved in DMSO. Optimal reactions were tested with the substrate naringenin (200 μM) at varying temperatures (20–70°C, 5°C increments) and pH values (5.0–9.0, 0.5 increments). The pH values were controlled using different buffers, such as acidic sodium citrate buffer (pH 5.0–6.5) and Tris/HCl buffer (pH 7.0–9.0). Different concentrations of the substrates, naringenin and isosakuranetin (0.2, 0.4, 0.8, 1.6, 3.2, 6.4, 12.8, 25.6, and 50 μM) were used to determine the kinetic parameters. Km and Vmax values were obtained using the Michaelis–Menten kinetics equation and non-linear regression analysis with GraphPad Prism 9 (GraphPad Software, La Jolla, CA, United States).

### Transient Overexpression Assay in Kumquat Peel Tissue

The transient overexpression assay was performed, as described previously (Wang F. et al., 2017; Gong et al., 2021; Zhao et al., 2021), with minor modifications. The ORF of each *FcFNSII* gene was inserted into the pBI121 vector using a seamless cloning strategy to generate the constructs. All constructs and the empty vector were separately introduced into the *Agrobacterium* strain, EHA105. The EHA105 strain was cultivated in liquid LB medium at 28°C; the cells were collected through centrifugation, and diluted to an OD_600_ of 0.8 in infiltration medium containing 0.05 M MES, 2 mM Na_3_PO_4_, 0.5% D-glucose, and 0.1 mM acetosyringone. The suspensions were injected into the peels of kumquat fruits (150 DAB). The injected fruits were maintained in the dark for 24 h and then under a long photoperiod (16 h light and 8 h dark) for 4 days. Flavonoid components were extracted, as described previously (Liu et al., 2018, 2020).

### Flavonoid Extraction and Ultra-High Performance Liquid Chromatography-Quadrupole Time-of-Flight-Mass Spectrometry Conditions

Filtered samples from enzyme assays and tissue sample extracts were separated on an ACQUITY UPLC BEH C18 column (2.1 × 100 mm, 1.7 μm, United Kingdom) connected to an ACQUITY UPLC I-Class PLUS System (Waters, Milford, MA). The mobile phases, consisting of 0.01% formic acid solution (A) and methanol (B) were used at a flow rate of 0.4 mL⋅min^–1^, with the following gradient program: 0–0.6 min, 90–80% A; 0.6–5 min, 80–30% A; 5–7 min, 30–10% A; and 7–8 min, 10–90% A. A photodiode-array detector was used to scan from 240 nm to 400 nm. A Xevo G2-S Q-TOF instrument (Waters MS Technologies, Manchester, United Kingdom) was used with an ESI source that was set from a mass: charge (m/z) ratio of 100–1,000. Data were collected in real time (scan time, 0.5 s; interval, 15 s) and processed using Waters UNIFI 1.7 software.

### Subcellular-Localization Assay

The full-length ORFs of *FcFNSII*s and their partial segments were used for subcellular-localization assays. These sequences (which lacked a stop codon) were separately ligated into the pBWA(V)HS-GLosgfp vector (BioRun, Wuhan, China) in frame with the 5′ end of the coding sequence of green fluorescent protein (GFP) to create different fusion constructs (i.e., 35S::FcFNSII-1/2-GFP, 35S::nFcFNSII-1/2-GFP, and 35S::delFcFNSII-1/2-GFP). The red fluorescent protein (RFP) gene was fused with the ER localization sequence (MKTNLFLFLFLIFSLLLSLSSAEF) and used as an ER-localization marker. The fusion constructs were co-transformed into tobacco leaves (*Nicotiana benthamiana*), along with the ER-localization marker, through *Agrobacteria*-mediated infiltration. Following transformation, the tobacco leaves were maintained at 25°C under a long photoperiod (16 h light and 8 h dark) for at least 48 h. The fluorescence signals were detected using an LSM 700 confocal microscope (Zeiss).

### Protein–Protein Interaction Assays

A split-ubiquitin yeast two-hybrid system (SU-YTH) in the DUAL membrane Kit 3 (Dualsystems Biotech, Zurich, Switzerland) was employed to test the potential interactions between FcFNSIIs and the upstream enzymes (i.e., FcCHS1, FcCHS2, FcCHI, and FcCHIL) in the flavonoid pathway. The interactions were assessed, as previously described (Waki et al., 2016; Fujino et al., 2018). Briefly, the ORF of FcFNSII-1 or FcFNSII-2 (without a translation-termination codon) was inserted into the *Sfi*I sites of the pBT3-SUC vector to express a recombinant protein with an N-terminal SUC peptide and a C-terminal Cub-LexA-VP16 protein, i.e., SUC-FcFNSII-1-Cub-LexA-VP16 or SUC-FcFNSII-2-Cub-LexA-VP16. The SUC peptide is a 19-residue signal peptide of yeast invertase (Suc2p). Cub-LexA-VP16 is a chimeric protein between the C-terminus of ubiquitin (Cub) and an artificial transcription factor (LexA-VP16). For upstream enzymes (referred to as X) involved in the flavonoid pathway, each of their ORFs was ligated into the *Sfi*I sites of the pPR3-N vector to generate recombinant proteins with a mutated N-terminal half of ubiquitin (NubG), i.e., NubG-X.

NMY51 yeast cells (Weidi Biotech, Shanghai, China) were transformed with one of the following pairs of plasmids (pBT3-SUC-FcFNSII-1/2-Cub-LexA-VP16 and pPR3-NubG-X; pBT3-SUC-FcFNSII-1/2-Cub-LexA-VP16 and pOst1-NubI as a positive control; pBT3-SUC-FcFNSII-1/2-Cub-LexA-VP16 and pPR3-NubG as a negative control). pOst1-NubI expresses a fusion protein comprising the yeast resident ER protein Ost1 and the wild-type N-terminal half of yeast ubiquitin (NubI). The transformed yeast cells were grown on synthetic dropout (SD) agar medium lacking tryptophan and leucine (SD/-WL), transferred to liquid SD/-WL medium, and cultured with agitation at 220 rotations/min for 12–16 h at 30°C. The cells were collected through centrifugation and diluted in ddH_2_O to an OD_600_ of 1.0. Five microliters of the yeast suspension, with five-fold serial dilutions in ddH_2_O, were grown on SD/-WL, SD/-WL lacking histidine (SD/-WLH), SD/-WLH lacking adenine (SD/-WLHAde), or SD/-WLHAde containing 1 mM 3-aminotriazole (SD/-WLHAde + AT). The cells were maintained at 30°C for approximately 3–4 days.

### Bimolecular Fluorescence Complementation Assay

For bimolecular fluorescence complementation assay (BiFC) analysis (Walter et al., 2004; Huang et al., 2015), the ORF of *FcFNSII-2* was cloned into the pSPYNE-35S vector (MiaoLing Plasmid Sharing Platform, Wuhan, China), containing the N-terminal sequence of yellow fluorescent protein (nYFP, AAs 1–174) to create the 35S::FcFNSII-2-nYFP fusion construct. In addition, each of three cytoplasmic flavonoid enzymes was recombined into the destination vector pSPYCE-35S (MiaoLing Plasmid Sharing Platform, Wuhan, China) containing the C-terminus of YFP (cYFP, AAs 175–239; i.e., 35S::FcCHS1/2/FcCHIL-cYFP). Tobacco leaves were co-infiltrated with an EHA105 strain harboring fusions containing the nYFP and cYFP fragments (Sparkes et al., 2006). YFP fluorescence was detected after 48 h to study subcellular localization.

### Accession Numbers

Sequence data for FcFNSII-1 (accession number sjg260860.1) and FcFNSII-2 (sjg260830.1) can be found at CPBD^1^.

## Results

### Identification and Sequence Analysis of Flavone Synthase II Homologs

**FIGURE 2:**
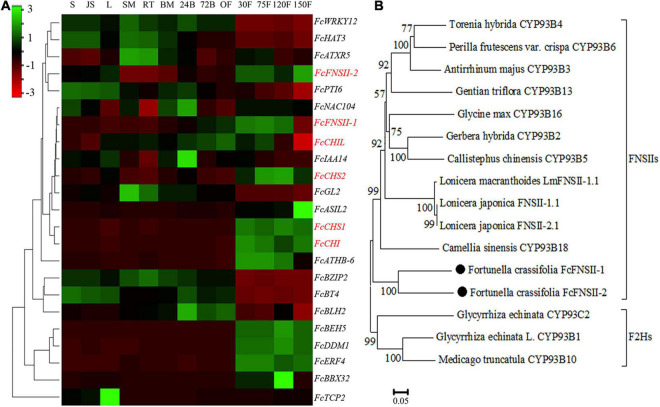
Identification of potential *FNSII* genes. **(A)** Heat map of the expression patterns of flavonoid biosynthesis-related genes using transcriptome data from 13 tissues of five organs. The color scale in the upper left corner represents log_2_-transformed counts normalized to the fragments per kilobase of transcript per million mapped reads. S, seed; JS, seedling; L, leaf; SM, stem; RT, root; BM, bud meristem; 24B, 24-h flower bud; 72B, 72-h flower bud; OF, opened flower; 30F, 30-day fruitlet; 75F, 75-day fruitlet; 120F, yellow-mature fruit (about 120 days); 150F, red-mature fruit (about 150 days). **(B)** A phylogenetic tree of FcFNSII-1, FcFNSII-2, and other functionally characterized FSNIIs and F2Hs was constructed using the neighbor-joining method with MEGA 6.0. The numbers at the nodes represent the bootstrap values from 1,000 replicates.

TBLASTN (*E*-value < 1e^–5^), using functionally characterized FNSIIs or F2Hs as queries against *Fortunella hindsii* genome^1^ and transcriptome (Zhu et al., 2019), identified many candidate genes. The AA sequence identity between the candidates and functionally characterized FSNIIs and F2Hs was low; therefore, it was difficult to identify the genuine F2Hs or FNSIIs among the candidates. We used transcriptome co-expression analyses to screen target genes. Based on transcriptome analysis of “Hongkong” kumquat (*F. hindsii*), generated from 13 different tissues from five organs (Zhu et al., 2019), two *FNSII* homologs were identified. Their expression was positively correlated with the expression levels of upstream genes in the flavonoid-biosynthesis pathway, such as *CHS1*, *CHS2*, *CHI*, and chalcone isomerase-like (*CHIL*) (Pearson’s correlation coefficient; between *FcFNSII-1* and flavonoid biosynthetic genes was >0.7 and between *FcFNSII-2* and flavonoid biosynthetic genes >0.6) (Figure 2A). The ORFs of the two *FNSII* homologs (*FcFNSII-1* and *FcFNSII-2*) were cloned through RT-PCR-based amplification from “Huapi” kumquat, a close relative of the “Hongkong” kumquat cultivar. The ORFs of *FcFNSII-1* and *FcFNSII-2* were 1542 and 1557 base pairs in length, encoding 513 and 518 amino acids with molecular weights (MWs) of 58.31 and 58.48 kDa, respectively. Sequence alignment revealed that both the FcFNSII-1 and FcFNSII-2 proteins harbor signature P450 motifs, such as a heme-binding motif and a Pro hinge region (Supplementary Figure 1). The phylogenetic analysis revealed that the proteins could be divided into two major groups (FSNIIs and F2Hs; Figure 2B). FcFNSII-1 and FcFNSII-2 clustered with members of the FNSII clade, which commonly catalyze the direct conversion of flavanones to flavones.

We analyzed the correlations between the accumulation of flavone derivatives and the expression patterns of *FcFNSII-1* and *FcFNSII-2* in different tissues including flowers, young leaves, young shoots, and fruit peels at 30, 90, and 150 DAB. Five flavone derivatives could be quantified in the different tissues of “Huapi” kumquats, including two *O*-glycosyl flavones (RHO and FOR) (Supplementary Figures 2A, 3) and three *C*-glycosyl flavones (APN, VIC, and MAR) (Supplementary Figures 2B, 3). The contents of *O*-glycosyl flavones in the peels at 30 DAB were higher than those in the other tissues and stages; *C*-glycosyl flavones showed preferential accumulation in flowers and in young leaves. Quantitative PCR was used to compare the spatiotemporal expression patterns of *FcFNSII*s (Supplementary Figure 2C) with the spatiotemporal-accumulation patterns of different flavone derivatives. The *FcFNSII-1* and *FcFNSII-2* transcripts were preferentially expressed in peels of fruits at 30 DAB, where the levels of *O*-glycosyl flavones were relatively high.

### *In vivo* and *vitro* Enzyme-Activity Assays for FcFNSIIs

**FIGURE 3:**
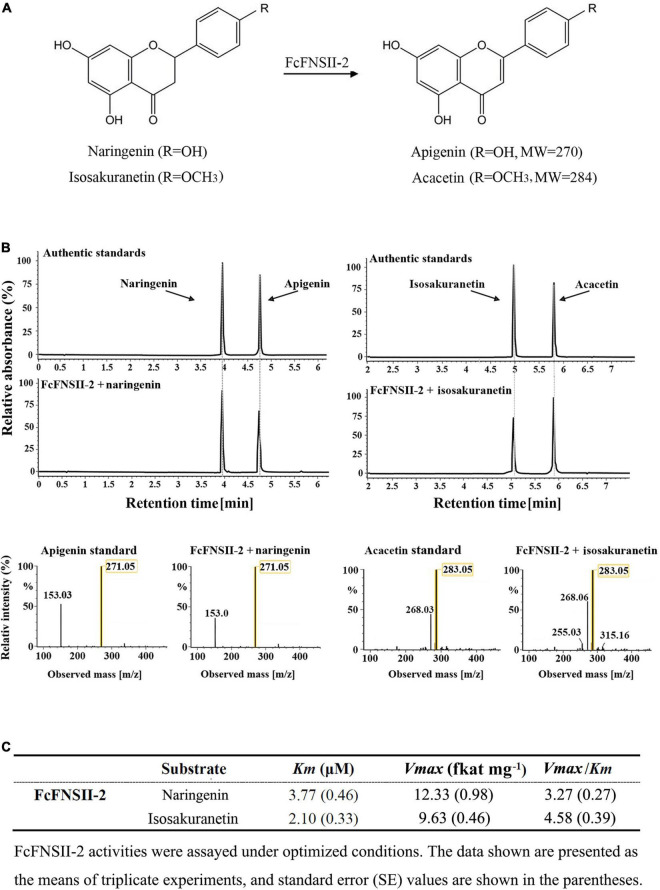
UPLC-Q-TOF-MS analysis of the reaction products of FcFNSIIs, as determined by performing *in vivo* enzyme-activity assays. **(A)** The schematic diagram represents the catalytic mechanism of FcFNSII-2. **(B)** *In vivo* enzymatic assays performed with recombinant FcFNSII-2. The assays were conducted using naringenin and isosakuranetin as substrates. The mass spectra of apigenin and acacetin standards and the reaction products produced by incubation with FcFNSII-2. **(C)** *In vitro* kinetic parameters of recombinant FcFNSII-2 were determined using yeast microsome extracts.

To examine the catalytic functions of both the *FcFNSII* genes, the ORF of each sequence was subcloned into the pESC-HIS vector and transformed into the WAT11 yeast strain. WAT11 cells express an *Arabidopsis* ATR1 P450 reductase that provides reducing equivalents to plant P450s, such as the FNSII enzymes. The transformed yeast cultures were incubated with naringenin or isosakuranetin. These two flavanones are the predominant precursors for the biosynthesis of flavone derivatives, such as RHO, FOR, isovitexin, vitexin, VIC, and MAR that are naturally present in kumquat fruits (Figure 1). After incubation for 24 h, yeast cells expressing *FcFNSII-2* metabolized naringenin and isosakuranetin to apigenin and acacetin, respectively (Figures 3A,B), whereas *FcFNSII-1* showed no detectable catalytic activities against naringenin and isosakuranetin. Control cells harboring the empty vector did not produce any detectable apigenin and acacetin. The reaction products were detected using UPLC-Q-TOF-MS; their retention times and mass-fragmentation patterns were compared with those of authentic standards.

To gain further insight into the enzymatic properties of FcFNSII-2, microsomes were extracted from the transformed yeast cells and assayed for their enzymatic activities on flavanone substrates in Tris/HCl buffer supplemented with NADPH (which supplied reducing equivalents for P450). After incubation for 1 h, the reaction products were extracted and analyzed using UPLC-Q-TOF-MS. Changing the pH or temperature strongly affected the activities of the recombinant enzymes; they exhibited maximum activity at pH 7.0∼7.5 and a temperature of 30∼35°C (Supplementary Figure 4). Under these optimized conditions, the kinetic parameters of FcFNSII-2 were determined. The Km and Vmax for naringenin were 3.77 μM and 12.33 fkat mg^–1^, and those for isosakuranetin were 2.10 μM and 9.63 fkat mg^–1^, respectively (Figure 3C). The relatively higher Vmax: Km ratio for isosakuranetin suggested that it was slightly more preferred than naringenin as an *in vitro* substrate for FcFNSII-2.

### *In planta* Enzyme Assays of Recombinant FcFNSIIs

**FIGURE 4:**
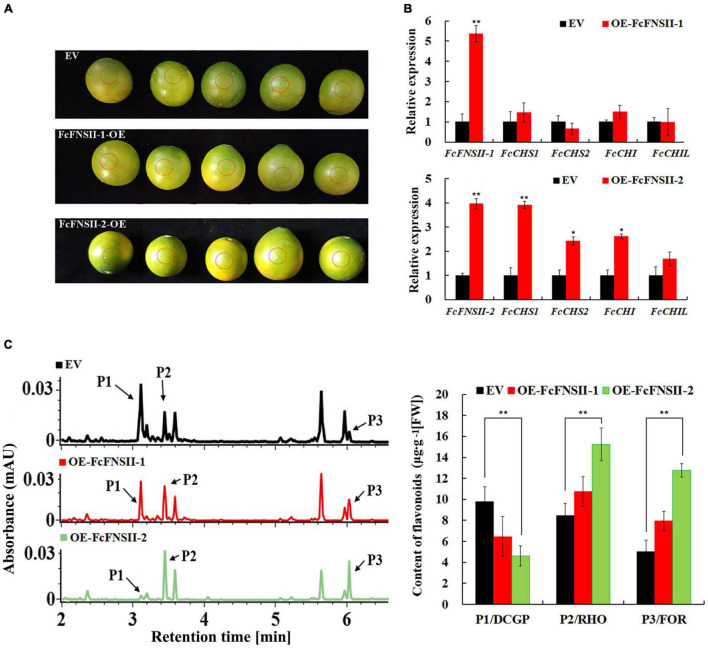
Transient over-expression of *FcFNSII*s in kumquat peels. **(A)** Harvested mature green fruits were infiltrated with pBI121 [empty vector (EV)] and pBI121-*FcFNSII*s (OE) in a climate-controlled chamber. The injection sites were circled by red dotted lines, where the peels were used to analyze gene-expression levels and flavonoid contents. **(B)** Expression of *FcFNSII*s and flavonoid biosynthesis-related genes in kumquat peels at 5 days after injection. **(C)** Measurements of flavonoids in kumquat peels at 5 days after injection. RHO, rhoifolin; FOR, fortunellin; DCGP, 3′,5′-di-C-β-glucopyranosylphloretin; FW, fresh weight. The data are presented as mean ± SE of five independent replicates. Asterisks indicate significant differences (*P < 0.05; **P < 0.01).

To further understand the metabolic functions of *FcFNSII*s *in planta*, we transiently overexpressed them in the peels of kumquat fruits at 150 DAB (Figure 4A), as done previously with success in citrus plants (Wang F. et al., 2017; Gong et al., 2021; Zhao et al., 2021). Gene-transcript levels at the injected sites were determined using quantitative PCR (Figure 4B). Compared to that in the control, the transcript levels of *FcFNSII*-1 and *FcFNSII*-2 were significantly higher at the corresponding injection sites. *FcFNSII-2* overexpression significantly enhanced the expression of *FcCHS1*, *FcCHS2*, *FcCHI*, and *FcCHIL* genes; however, *FcFNSII-1* overexpression did not significant affect the expression of these flavonoid-related genes. *CHS*, *CHI*, and *CHIL* are key enzymes in the early biosynthetic pathway of flavonoids, and their expression levels are closely related to flavonoid accumulation.

To confirm the flavonoid accumulation-enhancing function of *FcFNSII*s in kumquats, the main flavonoids were analyzed and quantified using UPLC-Q-TOF-MS at the injected sites. The three flavonoids, 3′,5′-di-*C*-β-glucopyranosylphloretin (DCGP), RHO, and FOR, showed differential accumulation between the control and pBI121-FcFNSII-2 vector-injected sites (Figure 4C and Supplementary Figure 3). The amounts of DCGP decreased significantly at the pBI121-FcFNSII-2 injected sites; however, those of RHO and FOR increased significantly. These changes were associated with the elevated levels of *FcFNSII-2* and other flavonoid-related genes. *FcFNSII-1* overexpression did not influence the flavonoid contents, as expected. Taken together, the results of *in vivo*, *in vitro*, and *in planta* experiments demonstrated that *FcFNSII-2* can directly convert flavanones into the corresponding flavones.

### Subcellular Localization

**FIGURE 5:**
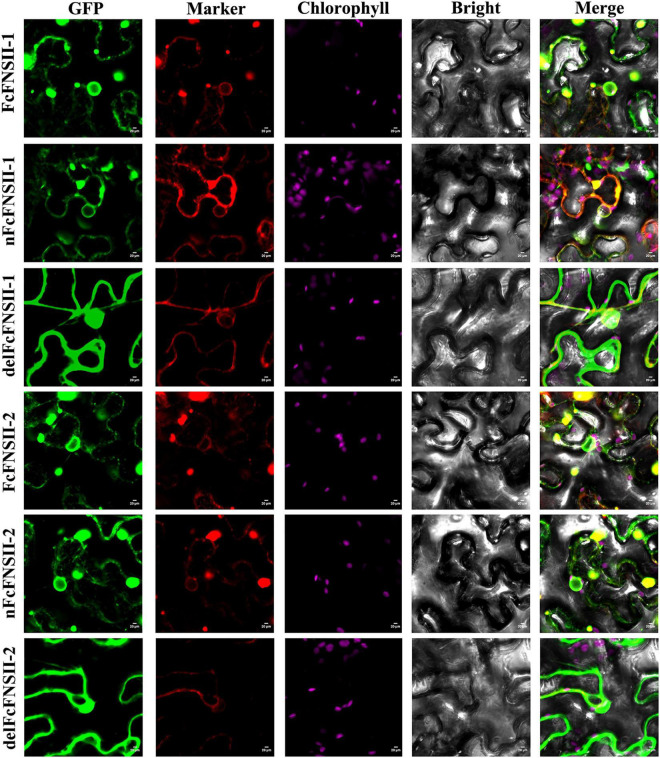
Subcellular localization of FcFNSIIs. Tobacco cells were transformed with vectors expressing different fusion constructs (FcFNSII-1-GFP, nFcFNSII-1-GFP, FcFNSII-2-GFP, or nFcFNSII-2-GFP) in combination with an ER-GFP marker. nFcFNSII-1-GFP and nFcFNSII-2-GFP comprise the first 27 residues of FcFNSII-1 and FcFNSII-2 fused to GFP, respectively. Co-localization of the fluorescent signals is apparent in the merged images, indicating that FcFNSII-1 and FcFNSII-2 localized to the ER. When tobacco leaves were transformed with delFcFNSII-1 (the remaining part of FcFNSII-1, AAs 28–513) fused to GFP or delFcFNSII-2 (AAs 28–518)-GFP in combination with a nucleocytosolic marker, fluorescent signals were detected in both the nucleus and the cytoplasm. Scale bars = 20 mm.

The two FcFNSIIs were predicted to harbor an N-terminal membrane-spanning domain (NMSD) spanning AAs 1–27 (Supplementary Figure 5), which anchors the P450s to the ER. The subcellular localizations of FcFNSII-1 and FcFNSII-2 were investigated by overexpressing C-terminally tagged GFP fusion proteins (FcFNSII-1-GFP and FcFNSII-2-GFP) to avoid interference with the NMSD. In tobacco leaf epidermal cells, the fluorescent signals of the fusion proteins were in the pattern of a network, which is consistent with the fluorescent signals specific to the ER (red color; Figure 5), suggesting that both FcFNSII-1 and FcFNSII-2 are localized to the ER, consistent with the localization of P450s. To examine whether this NMSD actually targeted FcFNSIIs to the ER, the NMSDs were expressed as GFP-labeled fusions (nFcFNSII-1-GFP and nFcFNSII-2-GFP); the localization pattern was consistent with the previously observed pattern. When FcFNSII-1 and FcFNSII-2 lacking the predicted NMSD were expressed as GFP-labeled fusions (delFcFNSII-1-GFP and delFcFNSII-2-GFP), the previously observed localization pattern was disrupted (Figure 5). Collectively, these results indicate that the predicted NMSD was necessary and essential for ER localization.

### Involvement of FcFNSIIs in the Flavone Metabolon

**FIGURE 6:**
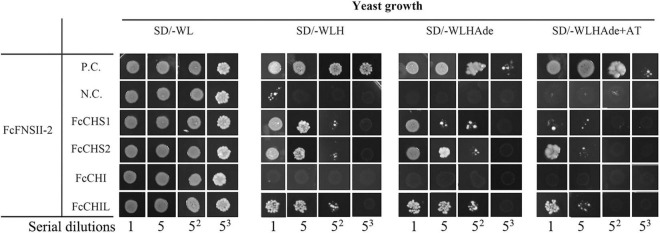
Interactions between FcFNSII-2 and upstream enzymes in the flavonoid pathway were assayed using a split-ubiquitin system. Yeast cells co-expressing SUC-FcFNSII-2-Cub-LexAVP16 with NubG fused to FcCHS1, FcCHS2, FcCHI, or FcCHIL. P.C. and N.C. refer to the positive control and negative control, respectively. For interactions between FcFNSII-1 and upstream enzymes, see Supplementary Figure 6.

The flavonoid-synthesizing enzymes in plants associate with the cytoplasmic surface of the ER to form metabolons, which is commonly mediated by ER-bound P450 (Shih et al., 2008; Waki et al., 2016; Fujino et al., 2018; Mameda et al., 2018). To examine whether FcFNSIIs interact with the upstream enzymes in the flavonoid pathway to form a complex, interactions between FcFNSII-1/FcFNSII-2 and CHS1, CHS2, CHI, and CHIL were analyzed using SU-YTH. Considering that FcFNSII enzymes contain an NMSD that locates them to the ER membrane, we designed SUC FcFNSII-Cub-LexA-VP16 constructs to ensure the NMSDs of both P450 proteins could be anchored to membranes. The yeast-growth results indicated that FcFNSII-2 interacted with FcCHS1, FcCHS2, and CHIL. No appreciable yeast growth was observed when interactions between FcFNSII-2 and CHI enzymes were assayed (Figure 6). In addition, no apparent interaction occurred between FcFNSII-1 and these enzymes (Supplementary Figure 6).

**FIGURE 7:**
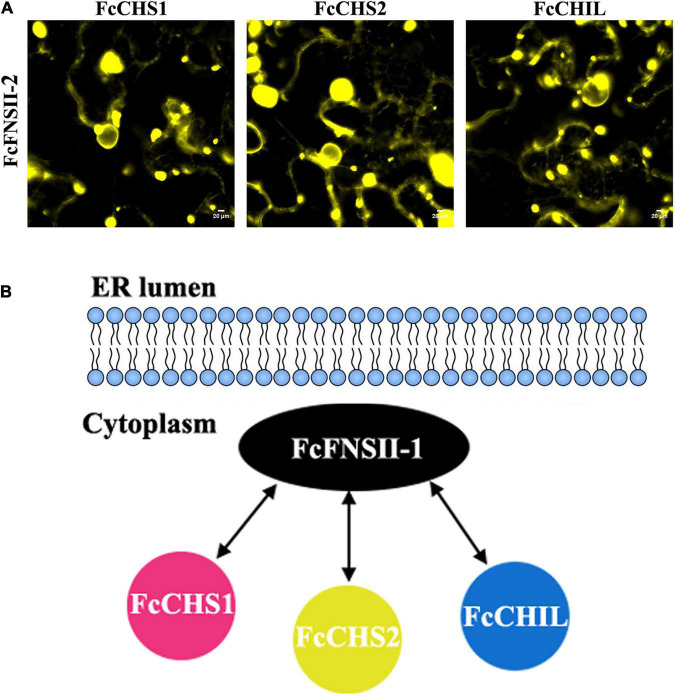
Detecting binary interactions between FcFNSII-2 and FcCHS1, FcCHS2, or FcCHIL by performing BiFC assays. **(A)** FcFNSII-2-nYFP was co-expressed with FcCHS1-cYFP, FcCHS2-cYFP, or FcCHIL-cYFP in tobacco leaf cells. Scale bars = 20 μm. **(B)** A suggested model of protein–protein interactions between FcFNSII-2 and FcCHS1, FcCHS2, or FcCHIL. FcFNSII-2 serves as a component of the kumquat flavone metabolon. Scale bars = 20 μm.

The binary protein-protein interactions between FcFNSII-2 and FcCHS1, FcCHS2, or FcCHIL were further examined *in planta* using BiFC assays. FcFNSII-2 was fused to the N-terminal end of a split YFP fragment (FcFNSII-2-nYFP) to preserve the ER-anchoring capacity, and other three enzymes were separately linked with a C-terminal split YFP fragment (FcCHS1-cYFP, FcCHS2-cYFP, or FcCHIL-cYFP). When FcFNSII-2-nYFP was transiently co-expressed with FcCHS1-cYFP, FcCHS2-cYFP, or FcCHIL-cYFP in tobacco leaf epidermal cells, the transformed cells generated yellow fluorescence signals (Figure 7), indicating that FcFNSII-2 interacted with FcCHS1, FcCHS2, and FcCHIL *in planta*.

## Discussion

The stepwise catalytic reactions of CHS and CHI drive carbon flow from the phenylpropanoid pathway toward the flavanone-biosynthesis pathways; these drive the formation of different subclasses of flavonoids. Functional loss of *Ruby* gene (which encodes a MYB transcription factor) caused by a single-nucleotide insertion that resulted in a frame-shift mutation abolished anthocyanin biosynthesis in kumquat plants (Butelli et al., 2017; Huang et al., 2018). Therefore, flavone-derived metabolites are one of the predominant flavonoids synthesized in kumquats. Flavone biosynthesis from flavanones was catalyzed by either FNSI or FNSII, which resulted in a C2–C3 double bond in the C-ring (Martens and Mithofer, 2005; Lee et al., 2008; Han et al., 2014; Lam et al., 2017).

Here, we investigated the activities of two kumquat FNSII homologs by transiently overexpressing the recombinant enzymes in kumquat peels and performing enzyme assays (Figures 3, 4). The catalytic activity of FcFNSII-2 was similar to that of gentian and soybean FNSII; it was capable of directly producing flavones from flavanones in yeast assays. FcFNSII-1 did not lead to flavone accumulation. Consistently, overexpressing *FcFNSII-2* in kumquats enhanced accumulation of the *O*-glycosyl flavones, RHO and FOR, which are naturally present in kumquat fruits. In licorice, flavones are synthesized from 2-hydroxyflavanones generated by GeFNSII, which functions as an F2H (Akashi et al., 1998). However, this catalytic activity could not be assigned to FcFNSII-2 due to the lack of 2-hydroxyflavanones accumulation, both *in vivo* and *in vitro*. Further studies are required to determine the involvement of *FcFNSII-1* in flavonoid biosynthesis. In addition, prior to functional characterization, the expression of FcFNSII-1 in yeast and plants should be verified. The success of P450 expression depends on factors such as the expression vectors, posttranslational modifications, compatibility with the host, and coupling efficiency with CPR.

Most flavones in kumquat fruits are predominantly present as *C*- or *O*-glycosides. Flavone *O*-glycosylation occurs after the flavone backbones are generated by FNSI and/or FNSII. To synthesize *C*-glycosyl flavones, flavanones are first converted into 2-hydroxyflavanones by F2Hs, the open-circular forms of which are subsequently *C*-glycosylated by CGTs, followed by dehydration to generate the corresponding *C*-glycosyl flavones (Brazier-Hicks et al., 2009; Du et al., 2010b; Falcone Ferreyra et al., 2013; Ito et al., 2017). Thus, two catalytic mechanisms for FNSII enzymes might exist in kumquat plants. F2H (CYP93G2) and FNSII (CYP93G1) enzymes are present in rice (Du et al., 2010a; Lam et al., 2014). The former channels flavanones to 2-hydroxyflavanones, which is a substrate for *C*-glycosylation by OsCGT (Brazier-Hicks et al., 2009; Du et al., 2010a). The latter converts flavanones to flavones for the biosynthesis of different tricin *O*-linked conjugates. CYP93G1 and CYP93G2 are key branch-point enzymes that compete for channeling flavanone substrates to flavones or 2-hydroxyflavanones, respectively. In this study, we observed such competition; the transient *FcFNSII-2* overexpression significantly elevated the levels of *O*-glycosyl flavones (RHO and FOR) and reduced the level of flavonoid *C*-glycoside (DCGP) (Figure 4). An intriguing question elicited by our findings is why DCGP has a significant reduction, but not *C*-glycosyl flavones. We speculated that the DCGP content is much higher than that of *C*-glycosyl flavones in kumquat peels. Consequently, When *FcFNSII-2* was overexpressed, the change in the amount of DCGP was easily discernable. However, owing to the low basal levels of *C*-glycosyl flavones, the changes in their levels were not obvious.

By expressing fusion constructs in tobacco leaf epidermis cells, we demonstrated that FcFNSII-1 and FcFNSII-2 were anchored to the ER (Figure 5). Several P450s involved in flavonoid biosynthesis play roles in lodging their respective metabolons to the cytoplasmic surface of the ER (Waki et al., 2016; Fujino et al., 2018; Mameda et al., 2018). In rice, protein–protein interaction analyses revealed that CHS1 interacted with flavonoid 3′-hydroxylase, flavanone 3-hydroxylase, dihydroflavonol 4-reductase, and anthocyanidin synthase 1, functioning as a platform for generating a flavonoid metabolon (Shih et al., 2008). This metabolon could be anchored to the ER via the ER-bound flavonoid 3′-hydroxylase. Soybean isoflavonoids are biosynthesized through the formation of dynamic metabolons anchored to the ER *via* two P450s, i.e., cinnamate 4-hydroxylase and 2-hydroxyisoflavanone synthase (Fliegmann et al., 2010; Dastmalchi et al., 2016; Waki et al., 2016; Mameda et al., 2018). Additionally, in snapdragon and torenia plants, ER-bound FNSII is a component of flavonoid metabolons (Fujino et al., 2018).

The protein–protein interaction studies (based on the SU-YTH system and BiFC assays, Figures 6, 7A and Supplementary Figure 6) demonstrated that ER-bound FcFNSII-2 was a component of the flavone metabolon and that it closely interacted with three upstream enzymes in flavonoid pathways, i.e., FcCHS1, FcCHS2, and FcCHIL (Figure 7B). Additionally, considering that FcFNSII-2 was preferentially expressed in young fruit peels (Supplementary Figure 2C), we speculate that this ER-bound metabolon likely plays a key role in biosynthesizing fruit-specific *O*-glycosyl flavones.

## Conclusion

We characterized a kumquat type II FNS (FcFNSII-2) that catalyzes the direct conversion of flavanones to flavones. *In vivo* and *vitro* assays showed that it could directly synthesize apigenin and acacetin from naringenin and isosakuranetin, respectively. Moreover, transient *FcFNSII-2* overexpression enhanced the transcription of structural genes in the flavonoid-biosynthesis pathway and drove the accumulation of different *O*-glycosyl flavones that are naturally present in kumquats. Subcellular-localization analyses and protein–protein interaction assays revealed that FcFNSII-2 was anchored to the ER and that it interacted with CHS1, CHS2, and CHIL. Our results provide strong evidence that FcFNSII-2 serves as a nucleation site for the *O*-glycosyl flavone metabolon that channels flavanones toward the biosynthesis of *O*-glycosyl flavones in kumquat fruits. These results would be useful for engineering the pathway to improve the composition of bioactive flavonoids in kumquat fruits.

## Data Availability Statement

The datasets presented in this study can be found in online repositories. The names of the repository/repositories and accession number(s) can be found in the article/Supplementary Material.

## Author Contributions

XGL and MZ designed the study. XGL, ST, and WX wrote the manuscript. ST, YY, CL, and XL performed the experiments. ST, YY, and XZ contributed to analyzing the results. All authors reviewed the manuscript.

## Conflict of Interest

The authors declare that the research was conducted in the absence of any commercial or financial relationships that could be construed as a potential conflict of interest.

## Publisher’s Note

All claims expressed in this article are solely those of the authors and do not necessarily represent those of their affiliated organizations, or those of the publisher, the editors and the reviewers. Any product that may be evaluated in this article, or claim that may be made by its manufacturer, is not guaranteed or endorsed by the publisher.

## Footnotes

1
http://citrus.hzau.edu.cn/index.php/

## Supplementary Material

Supplementary Figure 1 AA sequence alignment of FcFNSIIs with FNSII from other plants, comprising GhCYP93B2 of *Gerbera hybrida* (NCBI accession number AF156976), ThCYP93B4 of *Torenia hybrida* (AB028152), CsCYP93B18 of *Camellia sinensis* (FJ169499.1), LmFNSII-1.1 of *Lonicera macranthoides* (KU127580), LjFNSII-1.1 and LjFNSII-2.1 of *Lonicera japonica* (KU127576 and KU127578, respectively). The conserved motifs among P450s are boxed, including a region proline-rich membrane hinge (L/S/PPPS/G/TP), an I-helix (T/A/GAG/ATDTS/TA/S), a K-helix consensus sequence (KES/A/T/IL/FR), the PE/HRF consensus sequence, and a heme-binding domain (PFGS/TGRRG/A/SCPG).

Supplementary Figure 2 *O*/*C*-glycosyl flavone contents and *FcFNSII*-expression levels in kumquats. **(A)** Contents of *O*-glycosyl flavones in different kumquat tissues. RHO, rhoifolin; FOR, fortunellin; **(B)** Contents of *C*-glycosyl flavones in different kumquat tissues. VIC, vicenin-2; APN, 8-*C*-neohesperidosyl apigenin; MAR, margaritene; FW, fresh weight. **(C)** The expression levels of *FcFNSII-1* and *FcFNSII-2* were calculated via the 2^–ΔΔCt^ method. FL, flowers; YL, young leaves; YS, young shoots; 30P, peels of fruits at 30 DAB; 90P, peels of fruits at 90 DAB; 150P, peels of fruits at 150 DAB. The data are presented as the mean ± SE of three independent replicates. Asterisks indicate significant differences (^**^*P* < 0.01).

Supplementary Figure 3 Structure of key flavonoids in kumquat plants. DCGP, 3′,5′-di-*C*-β-glucopyranosylphloretin; APN, 8-*C*-neohesperidosyl apigenin; MAR, margaritene (8-*C*-neohesperidosyl acacetin); VIC, vicenin-2 (6,8-di-*C*-glucosylapigenin); RHO, rhoifolin (7-*O*-neohesperidosyl apigenin); FOR, fortunellin (7-*O*-neohesperidosyl acacetin).

Supplementary Figure 4 Effects of pH and temperature on the enzyme activities of FcFNSII-2 proteins. **(A)** Effects of pH on enzyme activities at 30°C for 1 h. Relative activities are presented as a percentage of the activity measured at pH 7.0 (100%). **(B)** Effects of temperature on enzyme activities at a pH of 7.0 for 1 h. Relative activities as a percentage of the activity measured at 30°C. The data are presented as the mean ± SE of three independent replicates.

Supplementary Figure 5 Detection of the NMSD of FcFNSII-1 and FcFNSII-2. Probabilities were calculated using the TMHMM server (TMHMM – 2.0 – Services – DTU Health Tech). The NMSDs of both FcFNSII-1 and FcFNSII-2 were predicted to be 26 AAs long.

Supplementary Figure 6 Interactions between FcFNSII-1 and upstream enzymes in the flavonoid pathway were assayed with a split-ubiquitin system. Yeast cells co-expressing SUC- FcFNSII-1-Cub-LexAVP16 with NubG-fused FcCHS1, FcCHS2, FcCHI, or FcCHIL.
