# Supplementary figures and images for: Functional Characterization of a Flavone Synthase That Participates in a Kumquat Flavone Metabolon
Source: Front Plant Sci. 2022 Mar 2;13:826780. doi: 10.3389/fpls.2022.826780 (PMC8924551; doi:10.3389/fpls.2022.826780)

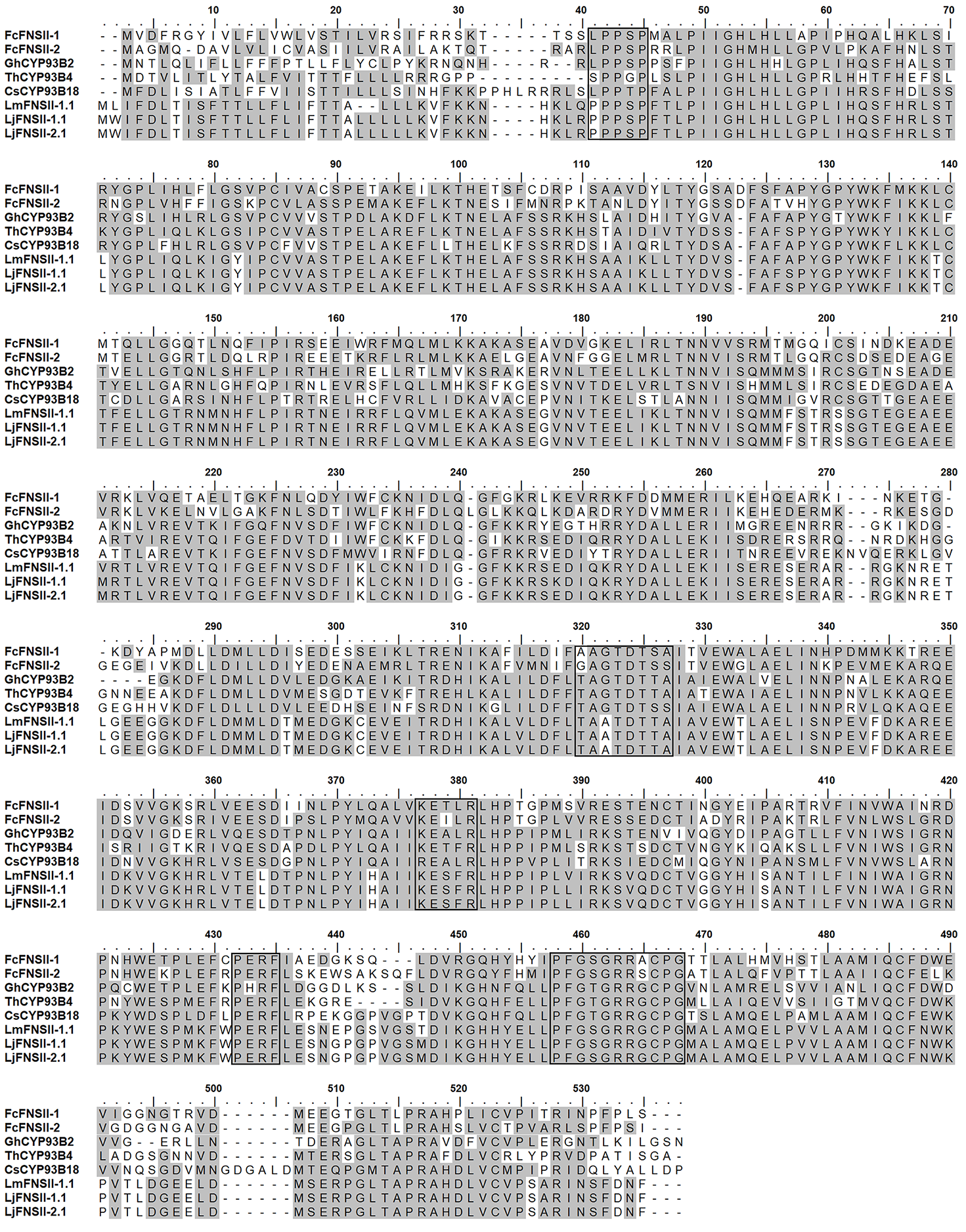

Supplement: Supplementary Figure 1 — AA sequence alignment of FcFNSIIs with FNSII from other plants, comprising GhCYP93B2 of Gerbera hybrida (NCBI accession number AF156976), ThCYP93B4 of Torenia hybrida (AB028152), CsCYP93B18 of Camellia sinensis (FJ169499.1), LmFNSII-1.1 of Lonicera macranthoides (KU127580), LjFNSII-1.1 and LjFNSII-2.1 of Lonicera japonica (KU127576 and KU127578, respectively). The conserved motifs among P450s are boxed, including a region proline-rich membrane hinge (L/S/PPPS/G/TP), an I-helix (T/A/GAG/ATDTS/TA/S), a K-helix consensus sequence (KES/A/T/IL/FR), the PE/HRF consensus sequence, and a heme-binding domain (PFGS/TGRRG/A/SCPG). [file Image_1.TIF]

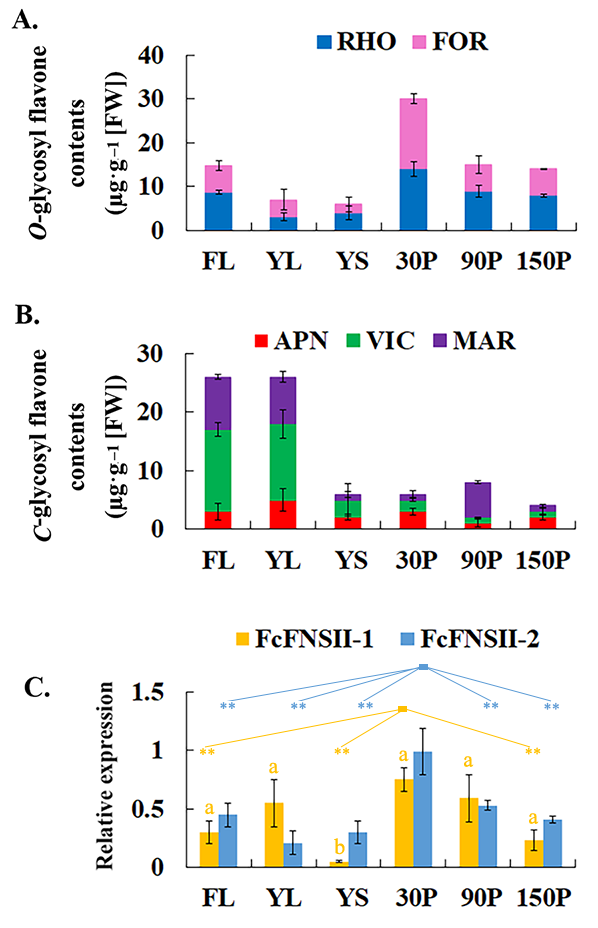

Supplement: Supplementary Figure 2 — O/C-glycosyl flavone contents and FcFNSII-expression levels in kumquats. (A) Contents of O-glycosyl flavones in different kumquat tissues. RHO, rhoifolin; FOR, fortunellin; (B) Contents of C-glycosyl flavones in different kumquat tissues. VIC, vicenin-2; APN, 8-C-neohesperidosyl apigenin; MAR, margaritene; FW, fresh weight. (C) The expression levels of FcFNSII-1 and FcFNSII-2 were calculated via the 2–ΔΔCt method. FL, flowers; YL, young leaves; YS, young shoots; 30P, peels of fruits at 30 DAB; 90P, peels of fruits at 90 DAB; 150P, peels of fruits at 150 DAB. The data are presented as the mean ± SE of three independent replicates. Asterisks indicate significant differences (**P < 0.01). [file Image_2.TIF]

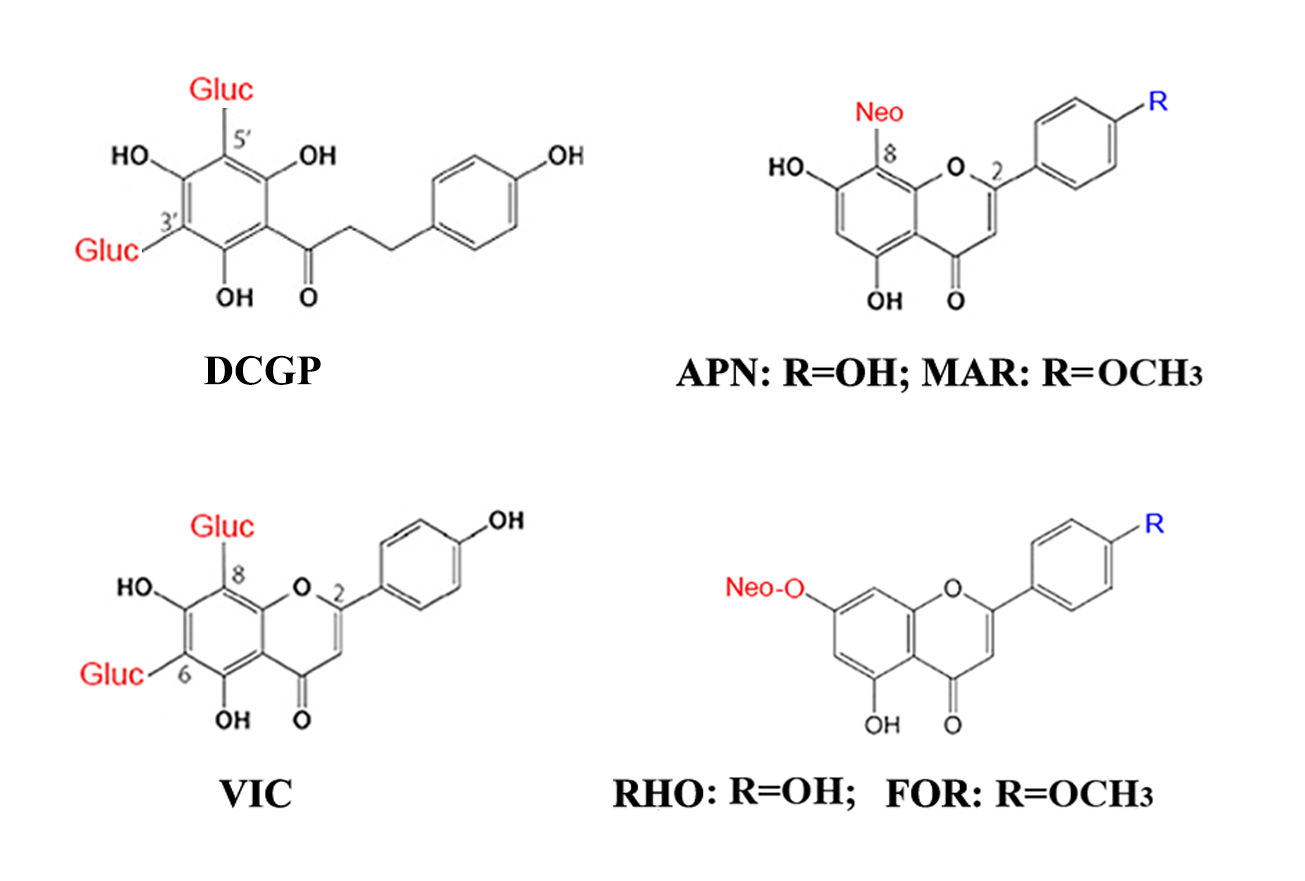

Supplement: Supplementary Figure 3 — Structure of key flavonoids in kumquat plants. DCGP, 3′,5′-di-C-β-glucopyranosylphloretin; APN, 8-C-neohesperidosyl apigenin; MAR, margaritene (8-C-neohesperidosyl acacetin); VIC, vicenin-2 (6,8-di-C-glucosylapigenin); RHO, rhoifolin (7-O-neohesperidosyl apigenin); FOR, fortunellin (7-O-neohesperidosyl acacetin). [file Image_3.TIF]

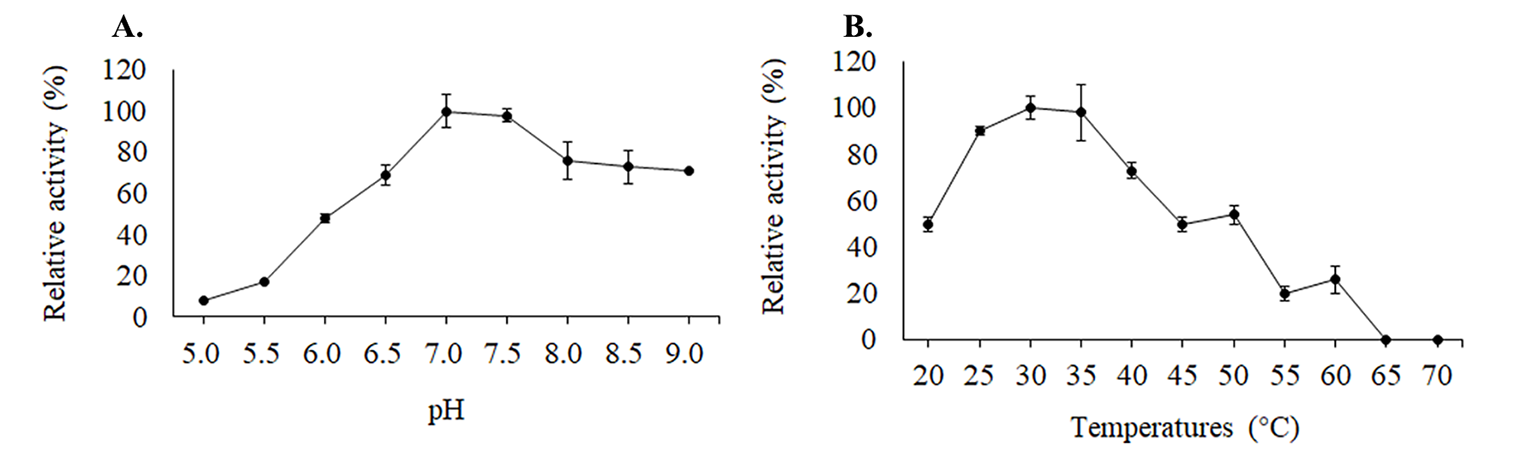

Supplement: Supplementary Figure 4 — Effects of pH and temperature on the enzyme activities of FcFNSII-2 proteins. (A) Effects of pH on enzyme activities at 30°C for 1 h. Relative activities are presented as a percentage of the activity measured at pH 7.0 (100%). (B) Effects of temperature on enzyme activities at a pH of 7.0 for 1 h. Relative activities as a percentage of the activity measured at 30°C. The data are presented as the mean ± SE of three independent replicates. [file Image_4.TIF]

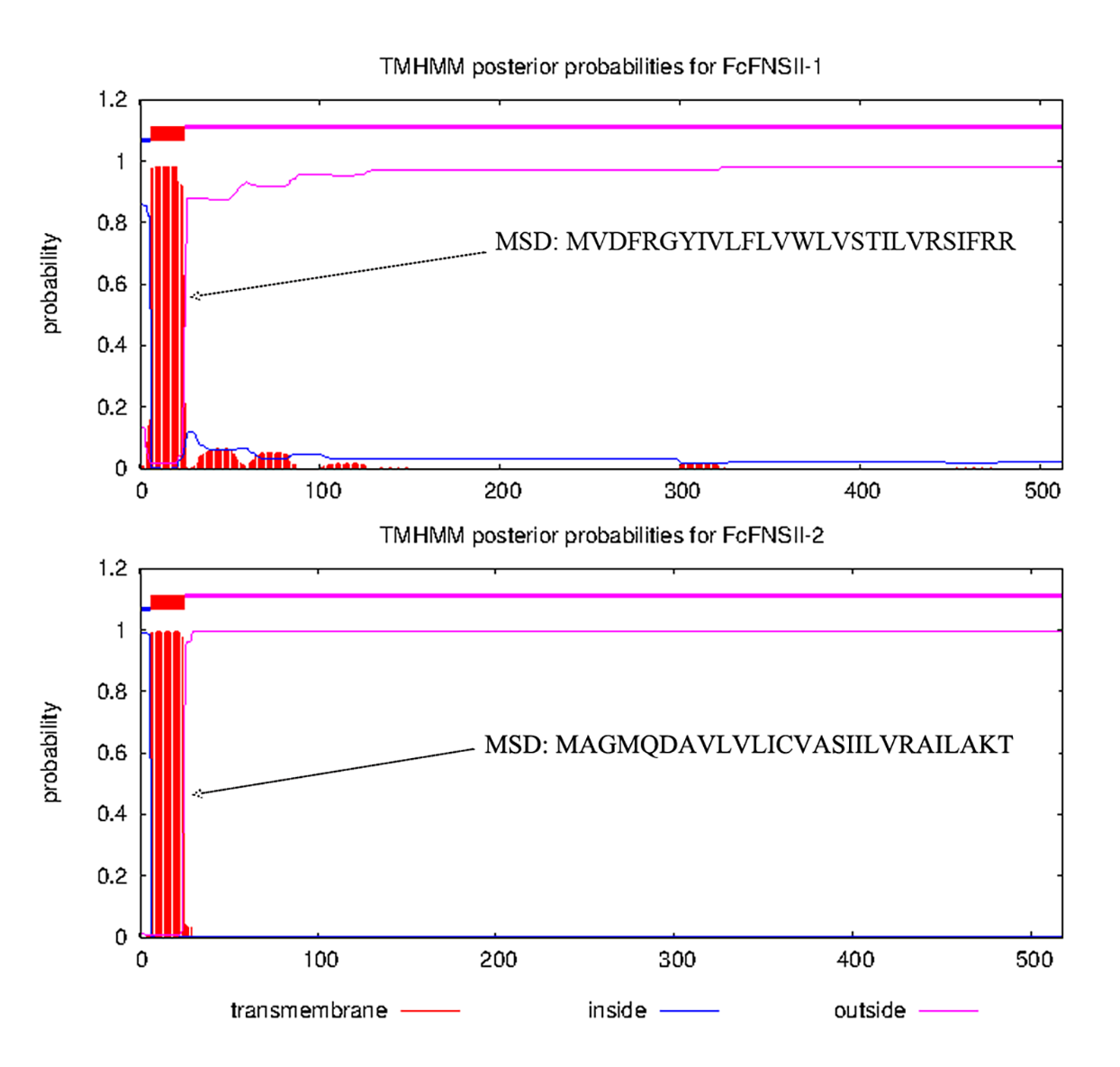

Supplement: Supplementary Figure 5 — Detection of the NMSD of FcFNSII-1 and FcFNSII-2. Probabilities were calculated using the TMHMM server (TMHMM – 2.0 – Services – DTU Health Tech). The NMSDs of both FcFNSII-1 and FcFNSII-2 were predicted to be 26 AAs long. [file Image_5.TIF]

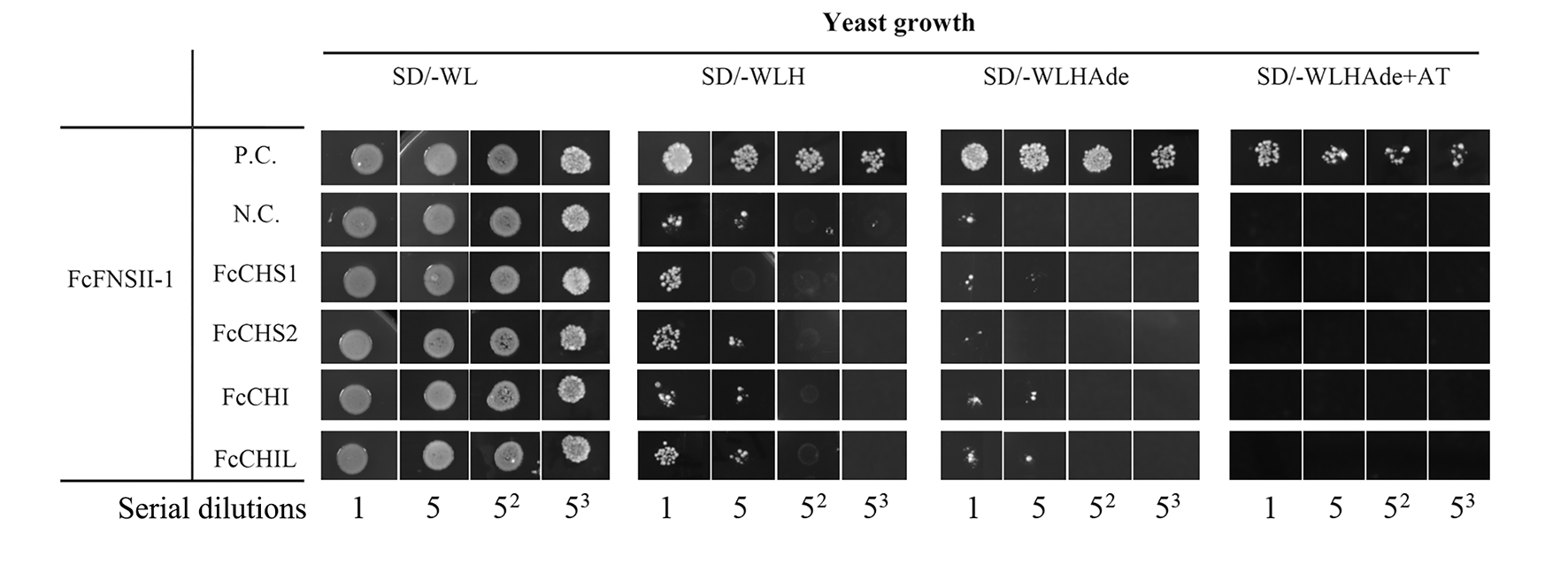

Supplement: Supplementary Figure 6 — Interactions between FcFNSII-1 and upstream enzymes in the flavonoid pathway were assayed with a split-ubiquitin system. Yeast cells co-expressing SUC- FcFNSII-1-Cub-LexAVP16 with NubG-fused FcCHS1, FcCHS2, FcCHI, or FcCHIL. [file Image_6.TIF]
