# Supplementary material for: Functional Characterization of a Flavone Synthase That Participates in a Kumquat Flavone Metabolon
Source: Front Plant Sci. 2022 Mar 2;13:826780. doi: 10.3389/fpls.2022.826780 (PMC8924551; doi:10.3389/fpls.2022.826780)
Supplement: Supplementary file 7 [file Table_1.DOCX]

**Table S1. List of primer sequences used in this article**

| **Primer names** | **Purpose** | **Primer sequences (5’-3’)** | |
| --- | --- | --- | --- |
|  |  | **Forward primer** | **Reverse primer** |
| *P_FcFNSII-1-ORF_* | ORF amplication | ATGGTTGATTTCAGAGGCTAC | TCAGGATAATGGAAACGGATTAATC |
| *P _FcFNSII-2-ORF_* |  | ATGGCTGGCATGCAAGATGC | TCAGATTGATGGGAATGGAC |
| *P _FcCHS1-ORF_* |  | ATGGTGACCGTCGATGAAG | TTAAGCAGCGGCAACACTGTG |
| *P _FcCHS2-ORF_* |  | ATGGCAACCGTTCAAGAGATC | TCAAGCTTTGATGGGGACAC |
| *P _FcCHI-ORF_* |  | ATGAATCCCTCACCGTCCGTC | TCATTTCATCTTATCACTAG |
| *P _FcCHIL-ORF_* |  | ATGGCCACTGAAGTTGTAATG | TCATTTTGATAACTCAGCTGAG |
| *P _FcFNSII-1-qPCR_* | qRT-PCR analysis | GCAAGATTATATTTGGTTCTG | CTCTGGTCAACTTGATTTCTG |
| *P _FcFNSII-2-qPCR_* |  | GGAGGGGAGCTTATGAGGTTGAC | CTGTCGCGAGCATCCTTAAGCTG |
| *P_Actin-qPCR_* |  | CCGACCGTATGAGCAAGGAAA | TTCCTGTGGACAATGGATGGA |
| *P_FcFNSII-1-YE_* | Yeast protein expression | AGGGCGGCCGCACTAGTATCGATGGTTGATTTCAGAGGCTAC | TTAATTAAGAGCTCAGGATAATGGAAACGGATTAATC |
| *P_FcFNSII-2-YE_* |  | ATGGCTGGCATGCAAGATGCAGGGCGGCCGCACTAGTATCG | TTAATTAAGAGCTCATCAGATTGATGGGAATGGAC |
| *P_FcFNSII-1-OV_* | Transient overexpression | CCGGGTGGTCAGTCCCTTATGGTTGATTTCAGAGGCTAC | AGGAGAGTTGTTGATTCAGGATAATGGAAACGGATTAATC |
| *P_FcFNSII-2-OV_* |  | CCGGGTGGTCAGTCCCTTATGGCTGGCATGCAAGATGC | AGGAGAGTTGTTGATTCATCAGATTGATGGGAATGGAC |
| *P_FcFNSII-1-gfp_* | Subcellular localization | CACGGGGGACTTTGCAACATGGTTGATTTCAGAGGC | TGAAGCGGCCGCTGTACAGGATAATGGAAACGGATTAATC |
| *P_nFcFNSII-1-gfp_* |  | CACGGGGGACTTTGCAACATGGTTGATTTCAGAGGC | TGAAGCGGCCGCTGTACATCTCCGGAAGATTGATCGG |
| *P_delFcFNSII-1-gfp_* |  | CACGGGGGACTTTGCAACATGAGTAAGACCACATCTAGTC | TGAAGCGGCCGCTGTACAGGATAATGGAAACGGATTAATC |
| *P_FcFNSII-2-gfp_* |  | CACGGGGGACTTTGCAACATGGCTGGCATGCAAGATGC | TGAAGCGGCCGCTGTACAGATTGATGGGAATGGAC |
| *P_nFcFNSII-2-gfp_* |  | CACGGGGGACTTTGCAACATGGCTGGCATGCAAGATGC | TGAAGCGGCCGCTGTACAAGTTTTGGCTAAGATGGCTCG |
| *P_delFcFNSII-2-gfp_* |  | CACGGGGGACTTTGCAACATGCAAACCAGGGCTCGCCTCCCG | TGAAGCGGCCGCTGTACAGATTGATGGGAATGGAC |
| *P_FcFNSII-1-SU_* | SU-YTH | ATGGCCATTACGGCCATGGTTGATTTCAGAGGCTAC | GGCCGAGGCGGCCTTGGATAATGGAAACGGATTAATC |
| *P_FcFNSII-2-SU_* |  | ATGGCCATTACGGCCATGGCTGGCATGCAAGATGC | GGCCGAGGCGGCCTTGATTGATGGGAATGGAC |
| *P_FcCHS1-SU_* |  | GTGGCCATTACGGCCATGGTGACCGTCGATGAAG | AGGCCGAGGCGGCGGCCGTTAAGCAGCGGCAACACTGTG |
| *P_FcCHS2-SU_* |  | GTGGCCATTACGGCCATGGCAACCGTTCAAGAGATC | AGGCCGAGGCGGCGGCCGTCAAGCTTTGATGGGGACAC |
| *P_FcCHI-SU_* |  | GTGGCCATTACGGCCATGAATCCCTCACCGTCCGTC | AGGCCGAGGCGGCGGCCGTCATTTCATCTTATCACTAG |
| *P_CHIL-SU_* |  | GTGGCCATTACGGCCATGGCCACTGAAGTTGTAATG | AGGCCGAGGCGGCGGCCGTCATTTTGATAACTCAGCTGAG |
| *P_FcFNSII-2-BiFC_* | BiFC assay | AGTGGATCCATCGATAGTATGGCTGGCATGCAAGATGC | CATCCCGGGAGCGGTACCGATTGATGGGAATGGAC |
| *P_FcCHS1-BiFC_* |  | AGTGGATCCATCGATAGTATGGTGACCGTCGATGAAG | CATCCCGGGAGCGGTACCAGCAGCGGCAACACTGTG |
| *P_FcCHS2-BiFC_* |  | AGTGGATCCATCGATAGTATGGCAACCGTTCAAGAGATC | CATCCCGGGAGCGGTACCAGCTTTGATGGGGACAC |
| *P_FcCHIL-BiFC_* |  | AGTGGATCCATCGATAGTATGGCCACTGAAGTTGTAATG | CATCCCGGGAGCGGTACCTTTTGATAACTCAGCTGAG |
